# Blood-letting in the Treatment of Pneumonia

**Published:** 1880-04

**Authors:** T. F. Worrell

**Affiliations:** Bloomington, Ills.


### ARTICLE II.

### BLOOD-LETTING IN THE TREATMENT OF PNEUMONIA. By T. F. WORRELL, M.D., of Bloomington, Ills.

The following paper on blood-letting was read by Dr. Worrell before the McLean County Medical Society:

*Gentlemen of the McLean County Medical Society:*

You asked me at your last meeting to submit to you at this time the views I entertain with regard to the agency of venesection in the control of pneumonia.

The first remark I have to make is that when I said I could demonstrate the potency of blood-letting to arrest that disease, I had reference only to the acute form of it, uncomplicated by any condition contraindicating antiphlogistics, only the sthenic variety, if we may so designate it.

I do not propose to introduce any authors for the purpose of showing that pneumonia is properly treated by bleeding, for this would not be germane to the inquiry. The specific subject assigned me requires me to show that the disease under consideration is treated *rationally*, when it is treated antiphlogistically, especially when it is treated by *venesection*.

I shall not enter upon the discussion of the question, "Are Congestion and Inflammation Different Entities?" but shall assume that the first stage of inflammation is congestion, which signifies an undue accumulation of blood in a part, that interferes with the onward movement of the sanguineous fluid. This stage of inflammation, which Andral calls hyperæmia, is marked by (of course I am considering active congestion) such inordinate activity of the part—of the lung in pneumonia—and by such exalted temperature as to induce not only functional disturbance, but also, if not arrested, serious deposits, especially of serous and plastic matter. In *all* cases of acute pneumonia, active congestion is a necessary antecedent of that morbid condition which gives rise to the effusions which render this disease one of so grave character. Without these deposits, pneumonia would be regarded as one of the mildest forms of disease. On account of the liability to their occurrence, it is classed among the gravest diseases in the great catalogue of human maladies. This is explicable upon the ground that during the period of simple engorgement, or congestion, the capillaries are easily emptied; and, getting rid of their redundant contents, readily assume their normal caliber, and so all the morbid and often fatal consequences which result from *established* inflammation are easily averted.

Having shown what I suppose none of you will deny, that pneumonia in its forming stage is attended by an increased amount of blood in the part which involves all the tissues that compose the pulmonary parenchyma, *i. e.*, the air cells, the minute tubes, the intervening cellular tissue, and the vascular ramifications, I will state, as the next condition, that the fibrin of the blood is in great excess, and that this excess of fibrin is accompanied by a corresponding excess of albumen or coagulable lymph; that these two substances unite to form that yellowish substance which we call the buffy coat, and that this composite substance indicates not only the intensity of the pulmonary lesion, but also the extent of it as well.

This buffy coat is not confined to those who are suffering from inflammation; it also abounds with those who are plethoric and with pregnant women; all from the same source, a predominance of fibrin in the blood. Andral says that "in nearly all cases of acute inflammation there is an excess of fibrin, and the lymph globules of the blood." That, from three parts in a thousand, which is the normal proportion it often rises to six and even ten parts in pneumonia and rheumatism. This great pathologist further says that the appearance of the buffy coat in pneumonia is dependent on this excess of fibrin, and that it materially affects both the duration and products of the local disease. [In this place allow me to digress so far as to say that there is no authority for the presence of this buffy coat (the evidence of such inflammation) in those diseases that are infections.]

Another position I assume is that in the incipiency of this disease there is always a *focus* from which the irritation radiates, that is that it is at first *local* and afterwards spreads out into a larger field, limited often by the confines of the organ which it assails. This original point of irritation serves to invite the blood to it and its neighborhood, which has the effect not only to intensify the fire already started but to extend its glow to the adjacent parts and thus, very soon, there is fuel enough for a general conflagration. The old aphorism, "Ubi irritatio ibi affluxus humorum," is as true to-day as it was when it was first uttered, the woeful defection from its indication of treatment to the contrary notwithstanding.

We now come to inquire what is the most urgent indication in the *treatment* of pneumonia, and *how* may that indication be most surely and promptly fulfilled?

I answer that the paramount indication is to relieve as quickly as possible the engorged vessels, and thus prevent the occurrence of those effusions which always render the disease a grave one.

As I would not throw combustibles into a fire, if I wished to extinguish it, so I would arrest that tendency to a rush of the fuel which fans and re-supplies the flames of inflammation towards the point at which it started, and thus expect to smother it before its flames have consumed the citadel of life. The blood, and that charged with an inordinate amount of the elements which promote inflammation, should not be permitted to circulate so rapidly and in such augmented quantity in the parts diseased. And this brings me to answer the second part of the query already propounded, viz., How may this indication be fulfilled? I answer that the most prompt, the most rational and the most certain way in which to accomplish it, is by reducing the inflammable material as fast as possible. The abstraction of blood directly from the general current is the only rational mode of arresting the flame already spreading, thus withdrawing those elements upon whose free supply pneumonia must depend—if I may so speak—for its progress towards that stage of it in which the deprivation of blood could be of no service, but which tend to curtail those resources upon which the patient might be compelled to rely, while he was waiting for those changes to take place, now rendered unavoidable by the establishment of that condition of the breathing organ which venesection could not possibly rectify.

I need not remind you, gentlemen, that all authors from the dawn of medicine down to a recent period, have advocated depletion by the lancet as the most prompt and reliable expedient for arresting pneumonia in its acute stage.

They have all insisted that this is demanded not for its direct effect arresting inflammation, but also for lessening the labor of the lungs and thus giving rest for the suffering organ, as far as it is possible to do that. They argue that as all the blood in the body must pass through the lungs, after reaching the heart, before it can be again distributed, and, as in pneumonia, a large portion of the lungs is incapable of performing its functions perfectly, an increased burden falls upon the healthy portion, and thus not only is the same part unduly loaded with blood, but the movements of respiration are necessarily accelerated. Against this old theory of pneumonia, as to its pathology and treatment, is arrayed the new doctrine that it is an infectious disease, that the local symptoms are but expressions of this infection which has tainted the whole body, and that, therefore, the patient should be sustained "ab initio" by such regimen and medicines as tend to overpower the infection and fortify the system against its ravages.

I believe that no standard work of American authorship has espoused this new theory; but it is adopted by some German and English pathologists. Before summing up what I have to say in the argument for venesection, I will briefly consider some objections to this modern idea.

And first, it places pneumonia in that class of diseases which are self-limited. There are but two distinct classes of infectious diseases, viz: those which are self-limited, and those which do not terminate spontaneously. To the first class, the disease under consideration must belong, if to either. Now, the general conviction is, that infectious diseases of this class are terminated by elimination of materies morbi which produces them, or its destruction by catalysis. Of this class typhoid fever may be regarded as the type. I think I may safely assume that there is no physician who comprehends the pathology of typhoid fever, who will say that it may be aborted or arrested in a time too short for elimination of the infectious matter. This is just as true of all other diseases of this character. They all have a definite course, and the limit of their duration depends upon the time required for their development, and for the destruction or elimination of that toxemic agent which induces them.

Now the proof that pneumonia does not belong to this infectious class of diseases, is to be found in the fact, first that there is no fixed period for its continuance, its duration being dependent not upon the occupation of the blood by a poison which it requires a certain number of days to remove, but that it may be easily arrested in its first stages, the inflammation terminating in complete resolution, and all in a few days ; and, secondly, that the products of the morbid condition of the lung, which we call pneumonia, are like those which result from inflammation existing in any part of the body, without any reference to the cause of that inflammation. Much more might be written on this subject, but I have already written enough. I will conclude this paper by reference to some reflections on the subject of it by Dr. Henry Hartshorne, of the University of Pennsylvania. He says that venesection is one of the oldest, and has been one of the most universal remedies for inflammation. Although blood-fearers have appeared now and then in all ages and nations, yet the aggregate testimony of the profession from Hypocrates down to the present time, has been in favor of blood-letting in the treatment of inflammations and congestions. In these later years it is true that bleeding has more opponents and fewer advocates than at any previous period in medical history. Why is this true ?

*First.* Because of reaction from the great abuse of the lancet.

*Second.* A radical change in the type of almost all diseases, by which they have become asthenic.

*Third.* False construction of recent science, which according to Virchow and others, makes no difference between morbid lesions whatever may be their cause ; and that stimulation, irritation, and inflammation are essentially and practically, as well as *causatively*, different degrees of the same vital impression.

*Fourth.* Leadership and fashion.

By the abstraction of blood in pneumonia we lessen—

1. The plethora of the vessels.
2. The number of red corpuscles.
3. The force of the heart's impulse.
4. The excitement of nervous system.

By all these influences venesection has the effect to diminish the vascular excitement which belongs to inflammation and thus diminish the liability to destructive disintegration. If this is true then the assumption of Prof. Bennet and others of his class, that inflammation is a self-limited process, which cannot be cut short, is certainly erroneous. What, in the last place, is the modus operandi of blood letting? I answer, it restores the equilibrium of the circulation; it reduces disproportionate vascular congestion; it restores the *balance* of the circulation. It absolutely relieves the organ inflamed of *that* which is an essential factor in inflammation, a sine qua non to its existence, *i. e.* an inordinate amount of blood, charged with an excess of those very elements which are so greatly concerned in the production of those consequences which alone make pneumonia a perilous disease.

